# Control of Strongyles in First-Season Grazing Ewe Lambs by Integrating Deworming and Thrice-Weekly Administration of Parasiticidal Fungal Spores

**DOI:** 10.3390/pathogens10101338

**Published:** 2021-10-17

**Authors:** Mathilde Voinot, Rodrigo Bonilla, Sérgio Sousa, Jaime Sanchís, Miguel Canhão-Dias, José Romero Delgado, João Lozano, Rita Sánchez-Andrade, María Sol Arias, Luís Madeira de Carvalho

**Affiliations:** 1 Control of Parasites Research Group (COPAR, GI-2120), Department of Animal Pathology, Faculty of Veterinary, University of Santiago de Compostela, 27142 Lugo, Spain; mat.vmei@gmail.com (M.V.); rita.sanchez-andrade@usc.es (R.S.-A.); mariasol.arias@usc.es (M.S.A.); 2 CARVAL Pharmaceuticals, Bogotá 110211, Colombia; rodrigo.bonilla@carval.com.co; 3 Vasco da Gama Research Centre (CIVG), Department of Veterinary Sciences, Vasco da Gama University School, Avenida José R. Sousa Fernandes 197 Lordemão, 3020-210 Coimbra, Portugal; ramalhosousa@gmail.com; 4 CIISA—Centro de Investigação Interdisciplinar em Sanidade Animal, Faculdade de Medicina Veterinária, Universidade de Lisboa, Avenida da Universidade Técnica, 1300-477 Lisbon, Portugal; miguel.dias.20@ucl.ac.uk (M.C.-D.); jlozano@fmv.ulisboa.pt (J.L.); 5 Parasitic Diseases, Veterinary Faculty, University of “La República (Regional Norte)”, Salto 50000, Uruguay; sanchisjaime@gmail.com; 6 Department of Geography, University College London, London WC1E 6BT, UK; 7 Veterinary Inspection, San Cristóbal de La Laguna City Hall, 38201 La Laguna, Spain; Jaromdel@lalaguna.es

**Keywords:** soil-transmitted helminths, ovine, biological control, *Mucor circinelloides*, *Duddingtonia flagrans*, EPG counts, hematic parameters

## Abstract

Parasiticidal fungi have been used in several in vivo experiments in livestock farms worldwide, constituting an effective tool for the biocontrol of gastrointestinal parasites in grazing animals. In the first year of study, two groups of eight first-season pasturing ewe lambs infected by strongyles were dewormed with albendazole, and then, the test group received an oral dose of 10^6^ chlamydospores of *Mucor circinelloides* and 10^6^ *Duddingtonia flagrans* individually and thrice a week from mid-September to May (FS1), while the control group remained without fungi (CT1). In the second year, two new groups of first-season grazing ewe lambs were treated with ivermectin and subjected to the same experimental design (FS2 and CT2, respectively). The anthelmintic efficacy was 96.6% (CT1), 95.6% (FS1), 96.1% (CT2), and 95.1% (FS2). The counts of strongyle egg output increased in the control groups (CT1 and CT2) throughout the study and reached numbers higher than 600 eggs per gram of feces (EPG), while in FS1 and FS2, they were <250 EPG. The values of red blood cell parameters registered for CT1 and CT2 were lower than those of the reference standards, while a significant increment was recorded in FS1 and FS2, and values within the physiological range were attained. It is concluded that integrating efficient anthelminthic deworming with rotational pasturing and the regular intake of chlamydospores of *M. circinelloides* and *D. flagrans* provides a helpful strategy for maintaining low levels of strongyle egg output in first-season grazing ewe lambs and improves their health status.

## 1. Introduction

Livestock management in pasturing regimes ensures the nutrition of the livestock but involves a risk of infection by different parasites affecting the gastrointestinal tract. The in-soil development of certain species of gastrointestinal parasites from oocysts and eggs passed in the feces of infected animals to the environment is responsible for the infection of herbivores while grazing [1]. Consequently, important clinical signs and lesions appear: namely, anemia, a reduction in the functional gastric gland mass, and intestinal villous atrophy, resulting in low productivity, important economic losses, and even high mortality rates [2]. For the purpose of reducing their impact, deworming is administered regularly, but any action on the free-living life stages is rarely considered. This situation frequently results in a notable misuse of efficient dewormers, i.e., incorrect dosing and/or increased frequency of administration, which can lead to the occurrence of anthelmintic resistance [3]. As a consequence, major threats to animal production are expected in the near future, and the situation seems likely to worsen due to the economic difficulties involved in the development of new anthelmintic drugs.

In recent decades, grazing systems have been significantly developed for the production of healthy food under an ecofriendly management, respectful toward the environment, where the use of chemicals tends to be reduced or has even disappeared [4]. Alternatives include grazing management strategies, such as pasture rotation and rest, or multispecies grazing [5], focused on reducing the exposure of animals to parasitic infective stages. Another option relies on destroying the endoparasites infecting sheep by the administration of tannin-rich plants or copper oxide wire particles [6,7,8]. Another possibility consists of interfering in the development of parasites in the soil, preventing some larvae from reaching their infective stage or by reducing their presence. Due to the transmission of strongyles that occurs through the ingestion of third-stage larvae (L3), certain soil saprophytic filamentous fungi capable of forming traps in their mycelium and capturing these mobile stages have been successfully tested. *Duddingtonia flagrans*, *Monacrosporium thaumasium,* and *Arthrobotrys oligospora* are the most frequently used larvicide species in in vivo trials worldwide [9,10,11,12]. Previous investigations showed a significant reduction in the risk of infection by strongyles in sheep given daily chlamydospores of *D. flagrans* during a period of 8–12 weeks [13,14]. At present, two formulations based on this fungus are commercially available: BioWorma^®^ and Bioverm^®^ [15,16].

In recent years, satisfactory results have been demonstrated in reducing fecal egg counts and improving the health status among livestock and captive wildlife species receiving, weekly, for periods longer than ten months, a blend of chlamydospores of the parasiticide fungi *Mucor circinelloides* (ovicide) and *Duddingtonia flagrans* (larvicide) [17,18,19]. The current in vivo study aimed to test this blend against strongyles affecting ewe lambs in a northern Spanish farm. For this purpose, fungal chlamydospores were given thrice a week during the grazing period (from September to May) to a group of first-season grazing ewe lambs previously dewormed with anthelmintic. The levels of strongyles eggs per gram (EPG), as well as the animals’ hematic parameters, were evaluated periodically.

## 2. Results

### 2.1. Anthelmintic Efficacy

Fecal analysis showed the presence of strongyle eggs throughout the study, belonging to genera *Trichostrongylus* (71%), *Teladorsagia* (63%), *Nematodirus* (29%)*, Chabertia* (25%)*,* and *Oesophagostomum* (15%). *Eimeria* spp. Oocysts, and *Moniezia benedeni* and *Trichuris ovis* eggs were identified sporadically; therefore, these parasites were not considered.

In the beginning of the first year (September), counts of 631 ± 201 and 638 ± 196 eggs per gram of feces (EPG) were observed in feces from the control (CT1) and test (FS1) groups, respectively; therefore, an anthelmintic treatment was administered. The numbers of EPG fourteen days later were 22 ± 32 (CT1) and 28 ± 32 (FS1), leading to an efficacy of 96.6% and 95.6%, respectively.

In the second year, strongyle egg output reached 637 ± 218 EPG in the control group (CT2) and 656 ± 203 EPG in the test group (FS2); fourteen days after the deworming, the EPG counts were 25 ± 238 and 31 ± 35, which indicates an efficacy of 96.1% and 95.1% in CT2 and FS2 groups, respectively.

### 2.2. Dynamics of Strongyle Egg Output

**Figure 1:**
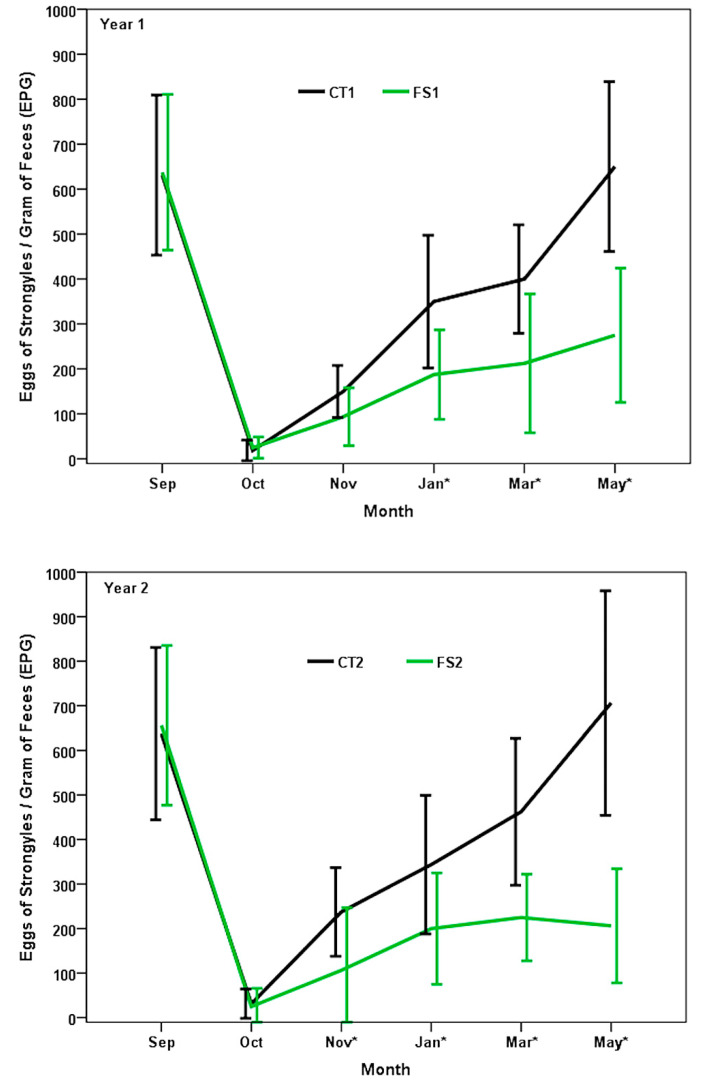
Dynamics of strongyle eggs per gram (EPG) of feces during the first-season grazing Latxa ewe lambs dewormed in September. CT: controls, not exposed to fungi; FS: provided a dosage of 10^6^ chlamydospores of *M. circinelloides* and 10^6^ chlamydospores of *D. flagrans*, thrice a week, from mid-September to May. * Month with a significant difference between EPG of FS and CT groups in both years of the field trials.

In the first year of the study, strongyle EPG in CT1 increased after the successful deworming (Figure 1), and values higher than 300 EPG were observed starting in the third month after treatment (m.a.t.). The EPG counts rose rapidly from this month onward, reaching values higher than 600 EPG at the eighth m.a.t. (May). In the ewe lambs of FS1, the EPG levels increased from the deworming (September) to the end of the study, although counts were always lower than 300 EPG. Significant differences between CT1 and FS1 were obtained from the third m.a.t. (*p* < 0.05).

Data obtained in the second year showed a pattern similar to the previous one (Figure 1), with a rapid increment in the EPG levels of CT2 group between the deworming and the end of the study; in the FS2 group, the average values of EPG did not exceed 250 during the same period. Significant differences between the two groups of ewe lambs were obtained from the second m.a.t. (November) to the end of the study.

Table 1 summarizes the values of fecal egg count reduction (FECR) and individuals positive coprology reduction (IPCR) throughout the study. In the first year, the amount of strongyle egg output halved at three m.a.t. in the lambs of the CT1 group, and counts close to the beginning of the study were attained at seven m.a.t. In the FS1 group, a significant reduction was observed during the trial, achieving 57% at the end (eight m.a.t.) (F= 5.491, *p* = 0.031). All the lambs of CT1 were positive by two m.a.t., whereas 75% of the lambs in FS1 achieved this between November and March.

Data collected in the second year were similar to those of the previous year. The values of strongyles EPG decreased by 46% at three m.a.t. in the CT2 group, whereas in the lambs given the fungal chlamydospores (FS2), the FECR was 70% in the same timeframe and 69% at the end of the study. Significant differences were obtained between the CT2 and FS2 groups (F = 5.491, *p* = 0.031). All the lambs of CT2 were positive by two m.a.t. and by four m.a.t. in FS1.

### 2.3. Variations in the Hematic Parameters

In the first year, the values of erythrocytes, hemoglobin, and hematocrit obtained after deworming in the CT1 group were lower than the reference standards (Table 2), whereas the number of white cells was similar until the end of the study and within the reference standards. In the lambs receiving the fungal spores (FS1), all the hematic parameters were within the reference values, with counts of red blood cell parameters higher than those of the CT1 group, and those of white blood cells lower than those of the controls. Significant differences were observed between the two groups throughout the study for the red blood cells, hemoglobin, mean cell volume, mean corpuscular hemoglobin, and mean corpuscular hemoglobin concentration.

At the beginning of the second year, the values of red blood cells parameters in both the CT2 and FS2 groups were lower than the reference counts (Table 3). After the anthelmintic treatment, CT2 red blood cells parameters increased briefly, then decreased to levels lower than the ones before the deworming. In contrast, the levels of red blood cell parameters increased in the FS2 group and attained the highest values, whereas the numbers of white blood cells decreased and reached the lowest numbers. Significant differences were obtained throughout the study except for the mean corpuscular hemoglobin concentration and the percentages of monocytes.

### 2.4. Combined Analysis of the Results Obtained during the Two Years

A similar dynamic of strongyle EPG was observed in the CT1 and CT2 groups, and no significant differences were shown (Z = −0.565, *p* = 0.572); the pattern of strongyle egg output was similar to that of the FS1 and FS2 groups (Z = −0.185, *p* = 0.854).

Significant differences were not obtained regarding the hematological parameters between the CT1 and CT2 groups, as well as between the FS1 and FS2 groups (*p* > 0.05).

A significant negative correlation was found between the numbers of strongyle egg output and the values of erythrocytes (ρ = −0.151, *p*= 0.036), hematocrit (ρ = −0.172, *p* = 0.017), mean corpuscular volume (ρ = −0.279, *p*= 0.001), and granulocytes (ρ = −0.305, *p* = 0.001). The correlations were positive for the EPG counts and the values of leukocytes (ρ = 0.190, *p* = 0.008) and lymphocytes (ρ = 0.301, *p*= 0.001).

## 3. Discussion

Despite many anthelmintic drugs being commercially available, the control of gastrointestinal parasites such as strongyles in grazing livestock remains a pending question due to the risk of rapid infection by infective L3 larval stages present in the soil and pasture [20]. With the objective of ensuring proper animal nutrition, rotational pasturing systems are often adopted [17,18]. In the current study, the deworming of first-season grazing ewe lambs with albendazole was successful (FECR > 95%), and five out of eight individuals did not pass eggs one month later. Treatment with ivermectin was also efficient, and half of the lambs were negative to coprological testing. These results are partly in agreement with previous investigations [21], although resistance to these anthelmintics has been reported [22].

The control of strongyles affecting grazing animals needs appropriate strategies to significantly reduce the risk of infection, and rotational pasturing is frequently advised. In the present investigation, lambs kept under a rotational system (15 days feeding and 45 days resting) passed eggs of strongyles one month after deworming, and we found egg output counts of higher than 300 EPG three months after, indicating that they were infected again very quickly. Considering that the development of L3 strongyles from eggs shed in the feces takes a minimum of seven days, grazing periods of 3–5 days appear necessary to reach successful results, though contradictory data have been reported [23,24]. Another inconvenience consists of the requirement of increasing the grassland area, as well as work hours and personnel [5,17].

With the aim of reducing the presence of the infective stages (L3) of strongyles, as well as their development in the soil, certain filamentous nematode-trapping fungi have been successfully assayed among different species of herbivores [14,25,26]. When sheep maintained stabled or under continuous grazing were provided chlamydospores of *D. flagrans*, the numbers of L3 larvae developing in the soil were decreased due to the trapping activity of this fungus [14,27], confirming this to be a very useful procedure to minimize the risk of infection while animals graze. Nevertheless, no significant effect on the egg output was recorded; thus, it appears very conceivable to bear in mind that the deworming of the sheep, together with the observation of preventive measures, could be highly valuable to their health status and the prevention of infection. By deworming with levamisole one group of sheep infected by gastrointestinal nematodes and providing handmade pellets with *D. flagrans* mycelium, low parasite burdens and better weight gains and hematocrit values were recorded [28]. In the current investigation, a beneficial effect was recorded in successfully dewormed ewe lambs maintained under rotational grazing and receiving (thrice a week) a blend of chlamydospores of *M. circinelloides* and *D. flagrans*. Low fecal levels of eggs of strongyles (<250 EPG) were observed, along with values of the red blood cell parameters higher than those in the controls (not given fungi) and within the reference standards. It should be noted that this is, to our knowledge, the first trial involving the administration of two fungi with complementary activity to grazing sheep over a long period. Based on herbivores being infected by ingesting immobile (oocysts, eggs, and metacercariae) or mobile (larvae) stages of the parasite when grazing, a complementary action appears very useful for reducing the possibilities of infection.

One interesting aspect relies on fungi with parasiticide activity, which do not survive in the soil beyond 2–3 months [29] because they are predated upon by other soil microorganisms, namely, bacteria, mites, other fungi, or even some species of nematodes. It is therefore essential to consider their regular administration to guarantee the expected results. Some studies demonstrated the viability of the manual or industrial elaboration of nutritional pellets for the administration of different dosages of fungal spores [14,30]. Another possibility consists of adding the spores during the manufacturing of energy blocks [9]. More recently, two flour-based formulations containing chlamydospores of *D. flagrans* in a very attractive and palatable feed supplement have been made commercially available in some countries, with notable results in reducing the contamination and, consequently, the infectivity of the pasture [15,16]. In the present research, milled cereal soaked with a liquid medium containing chlamydospores of *M. circinelloides* and *D. flagrans* was revealed to be very practical for the administration of these parasiticide fungi to ewe lambs. This procedure offers a highly efficient strategy for limiting the risk of infection by strongyles among pasturing animals and, consequently, the number of treatments required, thus constituting a sustainable solution that can be implemented for organic regimes. Another advantage is related to the commercial lifespan of commonly applied anthelmintics, such as benzimidazoles and macrocyclic lactones, which are preserved while avoiding their overuse.

## 4. Materials and Methods

### 4.1. Saprophytic Filamentous Fungi

Two strains of saprophytic filamentous fungi isolated from samples of soil and feces from livestock, *Mucor circinelloides* (CECT 20824) and *Duddingtonia flagrans* (CECT 20823), deposited at the Spanish Type Culture Collection (CECT, Valencia, Spain), were used in the current research. These strains were isolated by placing soil and fecal samples collected from different animal species in Galicia (NW Spain), onto Petri dishes with water agar and chloramphenicol and incubated at 25 °C for 15 days. Fungal isolates were subcultured in malt extract agar and corn meal agar. Monosporic cultures were obtained on potato glucose agar for morphometric and cultural characterization. Both *M. circinelloides* and *D. flagrans* were cultured in a submerged medium in the Laboratory of the COPAR Research Group (GI-2120; University of Santiago de Compostela, Spain) to obtain high numbers of chlamydospores [31,32].

### 4.2. Sheep

The present study was carried out in the farm “Sakona etxalde ekologikoa” (Azpeitia, Gipuzkoa, Basque Country, North Spain; 43°11′13.5”N 2°16′11.2”W), dedicated to the organic culture of vegetables and apple cider. Milk obtained from autochthonous Latxa sheep is commercialized as organic fresh packaged or used in the production of organic cheese.

Sheep are kept under a rotational grazing system, consisting of 15 days of feeding on mountain pastures and 45 days of resting. Supplementation is provided daily, and first-season grazing ewes receive individually ca. 200 g nutritional pellets/day.

### 4.3. Experimental Design

**Figure 2:**
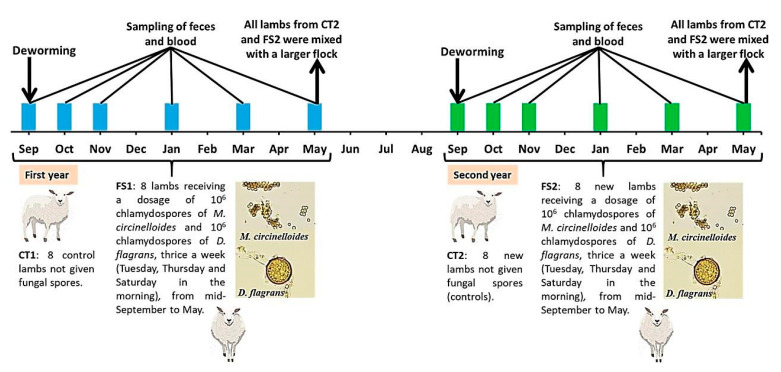
Design of the study involving first-season grazing Latxa ewe lambs maintained under a rotational pasturing regime from September to May, characterized by 15 days grazing and 45 days resting. For this purpose, a total of 10 fenced prairies of ca. 1 ha were divided into two lots. Every year, two groups were dewormed in September; CT: eight control lambs, not exposed to fungi; FS: eight lambs provided a dosage of 10^6^ chlamydospores of *M. circinelloides* and 10^6^ chlamydospores of *D. flagrans*, thrice a week, from mid-September to May. Each group grazed on five different grasslands without the possibility of sharing them. Different grasslands were utilized in the first and second years.

The current field trial was developed over two years, comprising first-grazing five-months old autochthonous Latxa ewe lambs. The analysis of feces in early September showed the presence of eggs of helminths, both in the first and second years; thus, deworming was provided, consisting of albendazole (7.5 mg/kg bw ALBENDEX 20^®,^ SP Veterinaria, Spain) orally in the first year and ivermectin subcutaneously in the second year (0.2 mg/kg bw NOROMECTIN 10^®^, Karizoo, Spain) (Figure 2). Rotation of dewormers was followed to prevent anthelmintic resistance [3].

Each year, a total of 16 lambs were sampled for 9 months, then mixed with a larger group at the end of the trial. In the second year, a total of 16 new lambs were sampled for 9 months and then mixed with the larger group also.

In the first year, the test group FS1 was formed by eight lambs receiving an approximate dosage of 10^6^ chlamydospores of *M. circinelloides* and 10^6^ chlamydospores of *D. flagrans*, three days per week (Tuesday, Thursday and Saturday in the morning), from mid-September to May. The control group CT1 was composed of other eight lambs that did not receive fungal spores. In the second year, a new group of new eight lambs was utilized as the test group (FS2) and provided with the chlamydospores as previously explained (three days per week from mid-September to May), whereas other new control group of eight lambs (CT2) remained without chlamydospores.

Every year, a total of 10 fenced prairies of ca. 1 ha were divided into two lots. Each group grazed on five different grasslands without the possibility of sharing them. From September to May, a rotational pasturing characterized by 15 days grazing and 45 days resting was implemented, in which a cycle was completed every 60 days (two months). From June onward, ewe lambs were managed jointly with the sheep (rams and ewes). Grasslands utilized in the first and second years were not the same.

With the aim of facilitating the administration of fungal spores to the lambs of FS1 and FS2, each year of the two allocated for the field trial, between September and May, the dosage was prepared in the lab. In brief, for every day of administration, 800 mL of liquid medium containing a blend of 10^4^ chlamydospores of *M. circinelloides* and 10^4^ *D. flagrans* / mL was added to 800 g milled cereal (wheat, barley and corn) and kept in a food dryer at 42 ºC until completely dry. The final product was mixed with 800 g nutritional pellets (1600 g in total), packaged into a plastic bag, kept at room temperature and shielded from sunlight, and sent monthly to the farm. Each lamb was provided individually 200 g of this formulation using feeders placed in each pasture for their supplementation with nutritional pellets. This 200 g/ewe/day of formulation provides a final concentration of 10^6^ spores of *M. circinelloides* and 10^6^ spores of *D. flagrans* per animal/day.

### 4.4. Coprological Analyses

For the investigation of the eggs of nematodes, fecal samples were individually collected throughout the study (Figure 2). Feces were analyzed by means of a quantitative flotation method (McMaster), consisting of 3 g feces of each sample homogenized in 42 mL water, and then the solution was filtered through a 150 µm mesh to two 12 mL filled tubes and centrifuged at 1500 rpm for 10 min. After discarding the supernatant, 10 mL saturated sodium chloride solution (gravity = 1.2) was added to the sediment and then observed in a McMaster chamber under a light microscope (Leica DM2500) at 10× [18,33].

With the objective of assessing the efficacy of the deworming, fecal samples taken the day of treatment and fourteen days later were analyzed, and the fecal egg count reduction (FECR) was calculated as follows:

FECR (%) = [1 – (EPG_day14_ / EPG_day0_)] × 100 (1)

Based on the guidelines enunciated by the World Association for the Advancement of Veterinary Parasitology (WAAVP), efficacy was considered to be achieved when FECRT > 95% [34].

By considering the usefulness of the information obtained in previous investigations [17,18], the reduction of the individuals positive to coprological tests (IPCR) was also estimated according to the formula:

IPCR (%) = [1 – (Nr positive ewes_day14_ / Nr positive ewes_day0_)] × 100 (2)

To gain more information about the different genera/species of the gastrointestinal nematodes affecting the sheep, fecal samples were cultured for 10–15 days at 25–27 ºC to allow the development of eggs to the third-stage infective larvae. Then, these L3 were collected by means of the Baermann procedure and identified according to morphological keys [33,35].

### 4.5. Blood Examinations

For the purpose of establishing the health status of the lambs, blood samples were collected by jugular venipuncture and examined by using an automated cell coulter counter (Vet Junior^®^, Diatron, Spain). Following the manufacturer’s instructions, the standard (physiological) values were considered to be 9–15 × 10^6^ red blood cells (RBC) / mL, 10–15 g hemoglobin (HB) / dL, 27–42% hematocrit (HCT), 24–32 fL mean corpuscular volume (MCV), 8–12 pg mean corpuscular hemoglobin (MCH), 32–42 g/dL mean corpuscular hemoglobin concentration (MCHC), 5–14 × 10^6^ white blood cells (WBC), 40–47% lymphocytes (LYM), 10–63% granulocytes (GRA), and 0–7% monocytes (MON).

### 4.6. Statistical Analyses

Using the Kolmogorov–Smirnov probe, the data collected for the fecal egg counts were found to be not normally distributed (Z = 2.309, *p* = 0.001), and the Levene test showed that the variances were not homogeneous (Statistic = 4.398, *p* = 0.031). Thus, a nonparametric Mann–Whitney U test was performed (significance level *p* < 0.05). The values of the hematological analyses were normally distributed, and then a one-way ANOVA was conducted (*p* < 0.05). The nonparametric Spearman’s rank test was used to evaluate the degree of correlation between the values of EPG and those of the hematological parameters (*p* < 0.05). All the probes were employed using the statistical package SPSS, version 21 (IBM SPSS, Inc., Chicago, IL, USA).

### 4.7. Institutional Collaboration

The research was performed within the framework of the agreement between the CIISA (Centre for Interdisciplinary Research in Animal Health, University of Lisbon, Portugal) and COPAR (Control of Parasites Group, GI-2120; University of Santiago de Compostela, Lugo, Spain). Additional people from different areas were involved in the authorial team of this research.

## 5. Conclusions

The data acquired in the current research led us to conclude that integrating deworming and the regular intake of chlamydospores of *M. circinelloides* and *D. flagrans* provides a helpful tool for maintaining low levels of strongyle egg output in first-season grazing ewe lambs and improves their health status. The adoption of this integrative approach has the potential to extend the commercial life of deworming products, reducing their administration and delaying the onset of anthelmintic resistance. The administration of the parasiticide fungi to the animals of the farm constitutes a green approach to parasite control, while also reducing chemical products and enhancing natural complementary/alternative biocontrol products. Finally, the culturing of these parasiticide fungi in a liquid medium and mixing it with milled cereal offers a very helpful procedure for facilitating the periodical administration of spores to pasturing animals and is also a sustainable solution to be implemented for organic production. Finally, it is necessary to emphasize the usefulness of providing grazing animals a mixture of chlamydospores of two filamentous fungi with ovicidal and larvicidal activity, because a complementary effect is expected based on the hyphae of *Mucor* which are able to break the eggshells, penetrate inside, and destroy the inner embryo, while traps are intercalated on the hyphae of *Duddingtonia* with the aim of capturing the larval stages. This formulation appears to also be a very helpful strategy for limiting the risk of mixture infections by different helminths as trematodes/ascarids and strongyles, and further research is underway to demonstrate it.

## Figures and Tables

**Table 1:** Values of fecal egg count reduction (FECR) and individuals positive coprology reduction (IPCR) in first-season grazing ewe lambs after deworming.

	Year 1				Year 2			
	FECR (%)		IPCR (%)		FECR (%)		IPCR (%)	
Month	CT1	FS1	CT1	FS1	CT2	FS2	CT2	FS2
Sep	Deworming				Deworming			
Oct	97	96	62.5	62.5	95	96	50	75
Nov	76	85	0	25	63	84	0	50 *
Jan	45	71*	0	25	46	70 *	0	0
Mar	37	67*	0	25	27	66 *	0	0
May	0	57*	0	0	0	69*	0	0

CT: control lambs; FS: lambs receiving a dosage of 10^6^ chlamydospores of *M. circinelloides* and 10^6^ chlamydospores of *D. flagrans*, thrice a week, from mid-September to May. ^*^ Significant difference between FS and CT groups in both years of the field trials.

**Table 2:** First year of study. Hematic parameters in first-season grazing Latxa lambs.

Reference Values		Red Blood Cell Parameters												White Blood Cell Parameters							
		RBC		HGB		HCT		MCV		MCH		MCHC		WBC		LYM		GRA		MON	
		9–15 10^6^/mL		10–15 g/dL		27–42%		24–32 fL		8–12 pg		32–42 g/dL		5–14 10^6^/mL		40–70%		10–63%		0–7%	
Month	Statistics	CT1	FS1	CT1	FS1	CT1	FS1	CT1	FS1	CT1	FS1	CT1	FS1	CT1	FS1	CT1	FS1	CT1	FS1	CT1	FS1
Sep	Mean	9.88	9.88	10.00	10.00	28.75	28.75	27.00	28.25	10.13	10.38	37.50	37.50	8.00	8.25	64.50	63.00	32.75	34.13	2.75	2.88
	SD	0.64	0.64	0.54	0.30	1.39	1.58	2.33	2.12	0.35	0.74	2.20	2.20	0.76	0.89	4.69	5.76	4.56	5.44	0.71	0.99
Oct	Mean	9.50	9.85	***9.80***	10.15	28.00	28.75	27.00	28.25	8.75	10.50 *	32.38	37.50 *	8.00	8.12	61.88	60.13	35.63	36.63	2.63	3.13
	SD	0.54	0.46	0.76	0.46	1.31	1.58	2.33	1.91	0.89	0.76	1.19	2.07	0.93	1.46	3.68	7.77	3.89	6.23	0.52	1.96
Nov	Mean	9.88	10.50	10.38	10.38	30.75	29.13	30.00	27.50	9.00	9.38	37.75	37.88	7.13	6.62	61.88	56.13	35.63	41.00	2.63	2.75
	SD	0.35	0.76	0.52	0.52	1.98	1.13	2.78	2.07	1.07	0.92	3.15	2.30	1.13	0.74	3.68	6.79	3.89	7.15	0.52	1.91
Jan	Mean	***8.75***	10.00 *	***9.75***	10.00	*26.63*	28.50	25.00	29.25	9.00	11.25 *	33.25	37.63 *	7.13	6.25 *	68.25	59.13 *	29.63	36.63 *	1.87	4.13 *
	SD	0.71	0.76	0.71	1.07	0.92	2.56	1.77	2.12	0.76	0.71	1.67	1.19	0.84	0.46	1.67	2.17	1.60	3.11	0.35	2.17
Mar	Mean	***8.75***	9.38	***9.50***	10.38 *	27.50	28.75	26.38	29.88 *	8.88	11.00 *	33.00	37.13 *	7.38	6.13 *	67.88	55.50 *	29.50	40.88 *	2.62	3.63
	SD	0.71	0.74	0.54	0.52	0.54	2.49	2.13	2.03	0.64	0.54	0.93	2.10	0.92	0.35	3.40	4.14	3.51	4.09	0.74	1.19
May	Mean	9.25	10.38 *	***9.88***	10.25	28.00	28.88	26.50	27.25	8.88	10.13 *	33.88	36.63 *	7.50	6.25 *	65.00	60.00 *	31.88	38.00 *	2.88	2.13
	SD	0.46	0.92	0.35	0.71	1.07	1.25	1.41	2.77	0.35	0.64	1.13	2.67	0.54	0.46	2.33	1.60	2.85	2.27	0.84	1.55
ANOVA	F =	22.429		8.672		2.086		8.255		55.479		29.801		7.326		35.109		30.157		4.129	
	*p* =	0.001		0.004		0.152		0.005		0.001		0.001		0.008		0.001		0.001		0.045	

CT: controls not receiving fungal spores; FS: lambs receiving a dosage of 10^6^ chlamydospores of *M. circinelloides* and 10^6^ chlamydospores of *D. flagrans*, thrice a week*,* from mid-September to May. RBC: red blood cells; HGB: hemoglobin; HCT: hematocrit; MCV: mean cell volume; MCH: mean corpuscular hemoglobin; MCHC: mean corpuscular hemoglobin concentration; WBC: white blood cells; LYM: lymphocytes; GRA: granulocytes; and MON: monocytes. *: Significant differences between CT1 and FS1. (Values lower than the reference standards are in red.)

**Table 3:** Second year of study. Hematic parameters in first-season grazing Latxa lambs.

		Red Blood Cell Parameters												White Blood Cell Parameters							
Reference values		RBC		HGB		HCT		MCV		MCH		MCHC		WBC		LYM		GRA		MON	
		9–15 10^6^/mL		10–15 g/dL		27–42%		24–32 fL		8–12 pg		32–42 g/dL		5–14 10^6^/mL		40–70%		10–63%		0–7%	
Month	Statistics	CT2	FS2	CT2	FS2	CT2	FS2	CT2	FS2	CT2	FS2	CT2	FS2	CT2	FS2	CT2	FS2	CT2	FS2	CT2	FS2
Sep	Mean	9.75	10.03	10.00	9.25	***26.25***	***25.38***	25.25	***24.88***	9.13	9.50	32.38	***31.25***	8.50	8.38	64.50	65.50	32.25	30.88	3.25	3.88
	SD	0.46	0.99	0.54	0.71	1.28	3.50	1.58	0.64	0.64	0.54	0.92	3.88	0.54	0.74	4.69	3.12	4.74	3.31	1.17	0.99
Oct	Mean	9.25	9.75	***9.63***	***9.25***	***26.88***	***25.63***	25.88	***23.88***	8.75	9.38	33.00	***30.88*** *	8.25	8.13	64.50	64.00	33.00	33.50	2.38	2.75
	SD	0.71	0.71	0.52	0.71	0.64	1.85	2.42	1.36	0.46	0.92	2.00	1.36	0.71	0.99	4.07	2.20	5.16	2.14	0.74	0.71
Nov	Mean	***8.75***	9.37 *	10.25	11.50 *	30.13	30.88	30.13	30.25	9.00	9.13	32.50	38.12	7.13	6.50 *	63.38	57.00 *	34.13	41.13 *	2.50	2.00
	SD	0.46	0.52	0.46	0.76	1.73	2.95	1.55	1.49	0.76	0.64	1.69	1.43	0.64	0.54	5.61	2.93	5.87	3.09	1.07	0.54
Jan	Mean	***8.88***	11.13 *	***9.88***	10.50 *	29.75	31.38	27.88	28.88	8.38	10.13 *	32.63	34.13	7.50	6.38 *	63.38	59.13	34.13	38.00	2.50	2.88
	SD	0.35	0.99	0.35	0.54	1.91	2.26	2.23	2.80	0.52	1.13	0.92	2.36	0.54	0.52	5.61	4.55	5.87	4.34	1.07	0.64
Mar	Mean	9.00	10.38 *	10.00	11.13 *	***26.75***	29.75 *	24.00	30.50 *	8.00	9.75 *	32.00	32.13	7.50	6.75	64.62	59.63 *	31.63	38.00 *	3.75	2.38 *
	SD	0.60	0.74	1.10	0.84	0.89	1.58	0.76	1.31	0.30	0.71	0.54	1.13	0.76	0.89	2.39	3.20	2.88	2.98	0.71	0.92
May	Mean	9.00	10.88 *	***9.75***	11.00 *	***26.75***	33.38 *	***23.88***	28.75 *	8.13	10.13 *	32.00	35.50 *	7.38	6.63 *	66.13	59.38 *	31.38	38.13 *	2.50	2.87
	SD	0.40	0.84	0.46	0.76	0.46	1.41	0.35	1.91	0.35	0.64	1.10	2.39	0.52	0.74	3.09	6.46	3.58	6.56	0.93	0.84
ANOVA	F	52.981		8.808		7.254		6.190		52.411		0.856		9.230		15.361		14.865		0.011	
	*p*	0.001		0.004		0.008		0.015		0.001		0.357		0.001		0.001		0.011		0.919	

CT: controls not receiving fungal spores; FS: lambs receiving a dosage of 10^6^ chlamydospores of *M. circinelloides* and 10^6^ chlamydospores of *D. flagrans*, thrice a week*,* from mid-September to May. RBC: red blood cells; HGB: hemoglobin; HCT: hematocrit; MCV: mean cell volume; MCH: mean corpuscular hemoglobin; MCHC: mean corpuscular hemoglobin concentration; WBC: white blood cells; LYM: lymphocytes; GRA: granulocytes; and MON: monocytes. *: Significant differences between CT2 and FS2. (Values lower than the reference standards are in red.)

## Data Availability

The data that support the findings of this study are available from the corresponding author, upon reasonable request.

## References

[1] Khadijah S., Kahn L., Walkden-Brown S., Bailey J., Bowers S. (2013). Soil moisture influences the development of *Haemonchus contortus* and *Trichostrongylus colubriformis* to third stage larvae. Vet. Parasitol..

[2] Charlier J., Morgan E., Rinaldi L., van Dijk J., Demeler J., Höglund J., Hertzberg H., Van Ranst B., Hendrickx G., Vercruysse J. (2014). Practices to optimise gastrointestinal nematode control on sheep, goat and cattle farms in Europe using targeted (selective) treatments. Vet. Rec..

[3] Furgasa W., Abunna F., Yimer L., Haile G. (2018). Review on anthelmintic resistance against gastrointestinal nematodes of small ruminants: Its status and future perspective in Ethiopia. J. Vet. Sci. Ani Husb..

[4] van den Pol-van Dasselaar A., Hennessy D., Isselstein J. (2020). Grazing of Dairy Cows in Europe—An in-depth analysis based on the perception of grassland experts. Sustainability.

[5] Kumar N., Rao T.K.S., Varghese A., Rathor V.S. (2012). Internal parasite management in grazing livestock. J. Parasit. Dis..

[6] Hoste H., Jackson F., Athanasiadou S., Thamsborg S.M., Hoskin S.O. (2006). The effects of tannin-rich plants on parasitic nematodes in ruminants. Trends Parasitol..

[7] Soli F., Terrill T., Shaik S., Getz W., Miller J., Vanguru M., Burke J. (2010). Efficacy of copper oxide wire particles against gastrointestinal nematodes in sheep and goats. Vet. Parasitol..

[8] Burke J.M., Miller J.E. (2020). Sustainable Approaches to Parasite Control in Ruminant Livestock. Vet. Clin. N. Am. Food Anim. Pr..

[9] Sagüés M.F., Fusé L.A., Fernández A.S., Iglesias L.E., Moreno F.C., Saumell C.A. (2011). Efficacy of an energy block containing *Duddingtonia flagrans* in the control of gastrointestinal nematodes of sheep. Parasitol. Res..

[10] Cai K.-Z., Wang F.-H., Wang K.-Y., Liu J.-L., Wang B.-B., Xu Q., Xue Y.-J., Wang F., Zhang C., Fang W.-X. (2017). In vitro predatory activity of *Arthrobotrys oligospora* and after passing through gastrointestinal tract of small ruminants on infective larvae of trichostrongylides. Exp. Parasitol..

[11] MendozCe-Gives P., López-Arellano M.E., Aguilar-Marcelino L., Olazarán-Jenkins S., Reyes-Guerrero D., Ramírez-Várgas G., Vega-Murillo V.E. (2018). The nematophagous fungus *Duddingtonia flagrans* reduces the gastrointestinal parasitic nematode larvae population in faeces of orally treated calves maintained under tropical conditions. Dose/Response assessment. Vet. Parasitol..

[12] Canhão-Dias M., Paz-Silva A., de Carvalho L.M. (2020). The efficacy of predatory fungi on the control of gastrointestinal parasites in domestic and wild animals—A systematic review. Vet. Parasitol..

[13] Gómez-Rincón C., Uriarte J., Valderrábano J. (2006). Efficiency of *Duddingtonia flagrans* against Trichostrongyle infections of sheep on mountain pastures. Vet. Parasitol..

[14] Marcelino L.A., Mendoza-De-Gives P., Torres-Hernández G., López-Arellano M., Becerril-Pérez C., Orihuela A., Torres-Acosta J.F.D.J., Olmedo-Juárez A. (2016). Consumption of nutritional pellets with *Duddingtonia flagrans* fungal chlamydospores reduces infective nematode larvae of *Haemonchus contortus* in faeces of Saint Croix lambs. J. Helminthol..

[15] Healey K., Lawlor C., Knox M.R., Chambers M., Lamb J. (2018). Field evaluation of *Duddingtonia flagrans* IAH 1297 for the reduction of worm burden in grazing animals: Tracer studies in sheep. Vet. Parasitol..

[16] Braga F.R., Ferraz C.M., da Silva E.N., de Araújo J.V. (2020). Efficiency of the Bioverm^®^ (*Duddingtonia flagrans*) fungal formulation to control in vivo and in vitro of *Haemonchus contortus* and Strongyloides papillosus in sheep. 3 Biotech.

[17] Hernández J.Á., Sánchez-Andrade R., Cazapal-Monteiro C.F., Arroyo F.L., Sanchís J.M., Paz-Silva A., Arias M.S. (2018). A combined effort to avoid strongyle infection in horses in an oceanic climate region: Rotational grazing and parasiticidal fungi. Parasites Vectors.

[18] Voinot M., Cazapal-Monteiro C., Hernández J.Á., Palomero A.M., Arroyo F.L., Sanchís J., Pedreira J., Sánchez-Andrade R., Paz-Silva A., Arias M.S. (2020). Integrating the control of helminths in dairy cattle: Deworming, rotational grazing and nutritional pellets with parasiticide fungi. Vet. Parasitol..

[19] Palomero A.M., Cazapal-Monteiro C.F., Valderrábano E., Paz-Silva A., Sánchez-Andrade R., Arias M.S. (2020). Soil fungi enable the control of gastrointestinal nematodes in wild bovidae captive in a zoological park: A 4-year trial. Parasitology.

[20] Vijayasarathi M.K., Sreekumar C., Venkataramanan R., Raman M. (2016). Influence of sustained deworming pressure on the anthelmintic resistance status in strongyles of sheep under field conditions. Trop. Anim. Health Prod..

[21] Seyoum Z., Demessie Y., Bogale B., Melaku A. (2017). Field evaluation of the efficacy of common anthelmintics used in the control of gastrointestinal nematodes of sheep in Dabat district, Northwest Ethiopia. Ir. Vet. J..

[22] Mondragon J., Olmedo-Juárez A., Reyes-Guerrero D.E., Ramírez-Vargas G., Ariza-Román A.E., López-Arellano M.E., De Gives P.M., Napolitano F. (2019). Detection of gastrointestinal nematode populations resistant to albendazole and ivermectin in sheep. Animals.

[23] Prasad M.S.R., Sundaram S.M., Gnanaraj P.T., Bandeswaran C., Harikrishnan T.J., Sivakumar T., Azhahiannambi P. (2019). Influence of intensive rearing, continuous and rotational grazing systems of management on parasitic load of lambs. Vet. World.

[24] Burke J., Miller J., Terrill T. (2009). Impact of rotational grazing on management of gastrointestinal nematodes in weaned lambs. Vet. Parasitol..

[25] Buzatti A., Santos C.D.P., Fernandes M.A.M., Yoshitani U.Y., Sprenger L.K., dos Santos C.D., Molento M.B. (2015). *Duddingtonia flagrans* in the control of gastrointestinal nematodes of horses. Exp. Parasitol..

[26] Malagón J.Á.H., Cazapal-Monteiro C.F., Quintero R.B., Salinero A.M.P., Torres M.I.S., Messnier M.V., Pena M.V., Blanco Á.R. (2019). Advantageous Fungi against Parasites Transmitted through Soil. Fungal Infection.

[27] Fontenot M., Miller J., Peña M., Larsen M., Gillespie A. (2003). Efficiency of feeding *Duddingtonia flagrans* chlamydospores to grazing ewes on reducing availability of parasitic nematode larvae on pasture. Vet. Parasitol..

[28] Vilela V.L.R., Feitosa T.F., Braga F.R., Vieira V.D., De Lucena S.C., De Araújo J.V. (2018). Control of sheep gastrointestinal nematodes using the combination of *Duddingtonia flagrans* and Levamisole Hydrochloride 5%. Rev. Bras. Parasitol. Vet..

[29] Saumell C., Fernández A., Echevarria F., Gonçalves I., Iglesias L., Sagües M., Rodríguez E. (2016). Lack of negative effects of the biological control agent *Duddingtonia flagrans* on soil nematodes and other nematophagous fungi. J. Helminthol..

[30] Hernández J.Á., Arroyo F.L., Suárez J., Monteiro C., Romasanta Á., López-Arellano M.E., Pedreira J., de Carvalho L.M.M., Sánchez-Andrade R., Arias M.S. (2016). Feeding horses with industrially manufactured pellets with fungal spores to promote nematode integrated control. Vet. Parasitol..

[31] Hernández J.A., Vázquez-Ruiz R.A., Cazapal-Monteiro C.F., Valderrábano E., Arroyo F.L., Francisco I., Miguélez S., Sánchez-Andrade R., Paz-Silva A., Arias M.S. (2017). Isolation of ovicidal fungi from fecal samples of captive animals maintained in a zoological park. J. Fungi.

[32] Viña C., Silva M.I., Palomero A.M., Voinot M., Vilá M., Hernández J.Á., Paz-Silva A., Sánchez-Andrade R., Cazapal-Monteiro C.F., Arias M.S. (2020). The Control of Zoonotic Soil-Transmitted Helminthoses Using Saprophytic Fungi. Pathogens.

[33] MAFF (1986). Manual of Veterinary Parasitological Laboratory Techniques.

[34] Geary T.G., Hosking B.C., Skuce P.J., von Samson-Himmelstjerna G., Maeder S., Holdsworth P., Pomroy W., Vercruysse J. (2012). World Association for the Advancement of Veterinary Parasitology (W.A.A.V.P.) Guideline: Anthelmintic combination products targeting nematode infections of ruminants and horses. Vet. Parasitol..

[35] VanWyk J., Cabaret J., Michael L.M. (2004). Morphological identification of nematode larvae of small ruminants and cattle simplified. Vet. Parasitol..

